# Supplementary material for: Secreted protease PRSS35 suppresses hepatocellular carcinoma by disabling CXCL2-mediated neutrophil extracellular traps
Source: Nat Commun. 2023 Mar 18;14:1513. doi: 10.1038/s41467-023-37227-z (PMC10024721; doi:10.1038/s41467-023-37227-z)

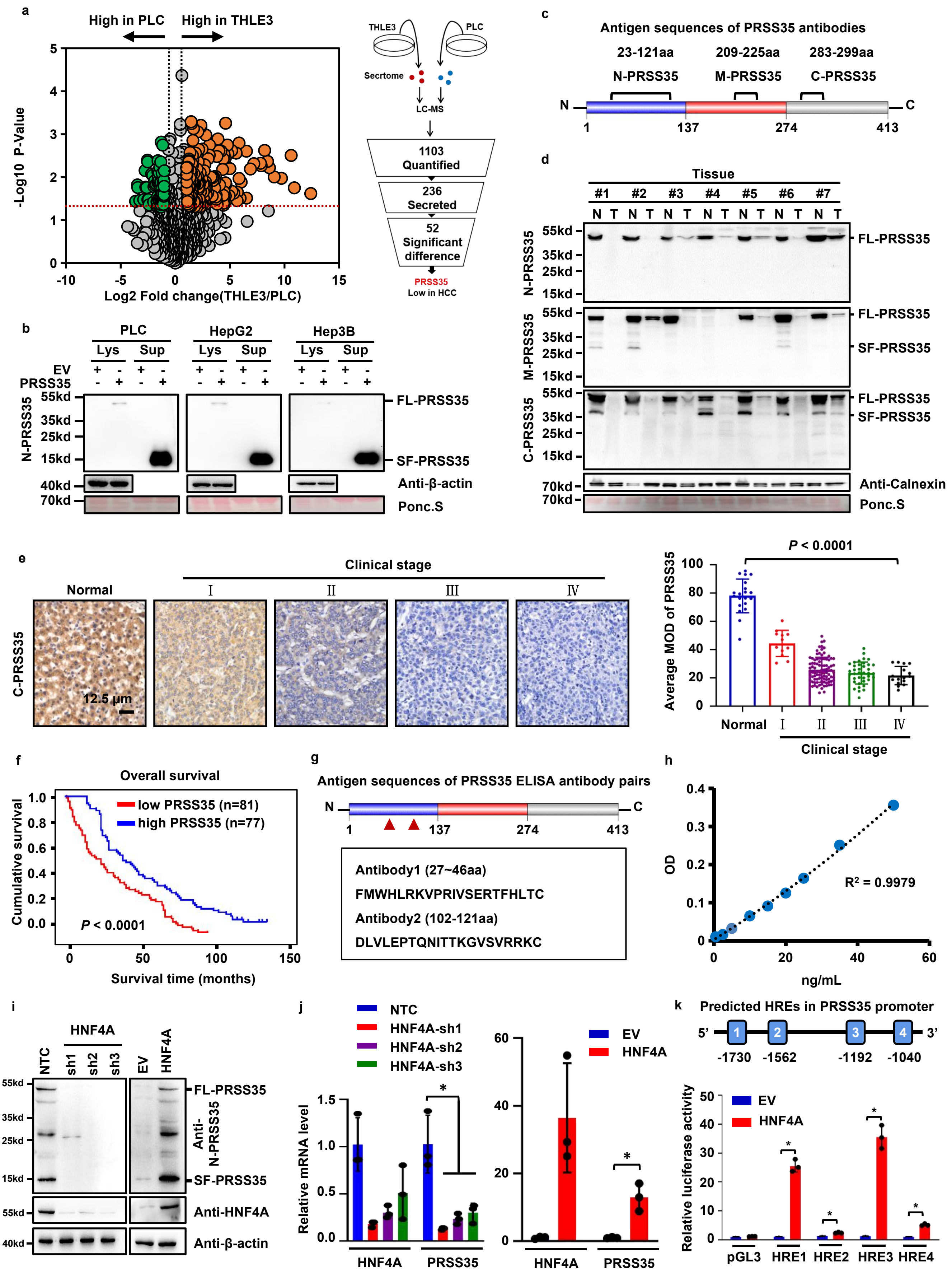

Supplementary Figure 1

Supplementary Fig. 1. PRSS35 is a secreted protein that decreased in HCC patients

a, Proteomic analysis and comparison of THLE3 and PLC secretome (with non-classical secreted proteins) presented as a volcano plot. The red transverse dashed line indicates adjusted *P*-value of 0.05. The left black longitudinal dashed line indicates a fold-change (FC) of 0.5 and the right black longitudinal dashed line indicates a FC of 2.0. Orange dots: significantly increased proteins in THLE3 secretome ($P < 0.05$, FC > 2.0). Green dots: significantly decreased proteins in THLE3 ($P < 0.05$, FC < 0.5). The experiment was repeated three times (left panel). Diagram of the screening strategy for PRSS35 being the most significantly decreased secreted protein in HCC (right panel)

b, Western blot analysis of intracellular (Lys: lysate) and extracellular (Sup: supernatant) PRSS35 protein levels with anti-N-PRSS35 antibody in PLC, HepG2 and Hep3B cells stably expressing PRSS35 or EV. Ponceau staining and β-actin served as loading control.

c, Schematic diagram of the antigen sequences of three PRSS35 antibodies.

d, PRSS35 protein levels were determined with three different antibodies by western blot using the paired human HCC tissues (T) and adjacent non-cancerous liver tissues (N). Ponceau staining and calnexin served as loading control. FL: full length. SF: short form.

e, Representative IHC images of PRSS35 staining in normal liver tissues (normal) and HCC specimens of different clinical stages I-IV (healthy donors, n= 20; patients with HCC, stage I (n= 12), II (n= 92), III (n= 38) and IV (n= 16)). Antibody against C-PRSS35 was used (left panel). Statistical quantification of the mean optical density (MOD) values for PRSS35 staining in IHC assay (right panel).

f, Kaplan-Meier curves with univariate analyses of patients with low versus high PRSS35 expression.

g, Schematic diagram of the antigen sequences of antibody pairs used in ELISA kit.

h, The standard curve of PRSS35 ELISA kit. We set ten diluted concentrations of purified PRSS35 domain1 protein fragment and determined the corresponding OD450 to calculate the linearity information.

i, Western blot analysis of PRSS35 protein when knocking down HNF4A with different shRNAs or overexpressing HNF4A in HepG2 cells. β-actin served as a loading control. FL: full length. SF: short form

j, Quantitative real-time PCR analysis of PRSS35 mRNA levels when knocking down HNF4A with different shRNAs or overexpressing HNF4A in HepG2 cells (n=3 biologically replicates).

k, A diagram shows the sites and sequences of potential HNF4A responsive elements (HREs) in *PRSS35* gene (upper panel). Luciferase assays were performed to identify HREs in *PRSS35* gene (lower panel, n=3 biologically replicates).

Data are presented as the mean ± s.e.m. (e) or ± s.d (j, k). Statistical significance was determined by two-tailed unpaired Student's t-test (a, e) or log-rank test (f, j ,k). The blotting experiments were repeated at least three times with biological replicates (b, d, i). Source data are provided as a Source Data file.

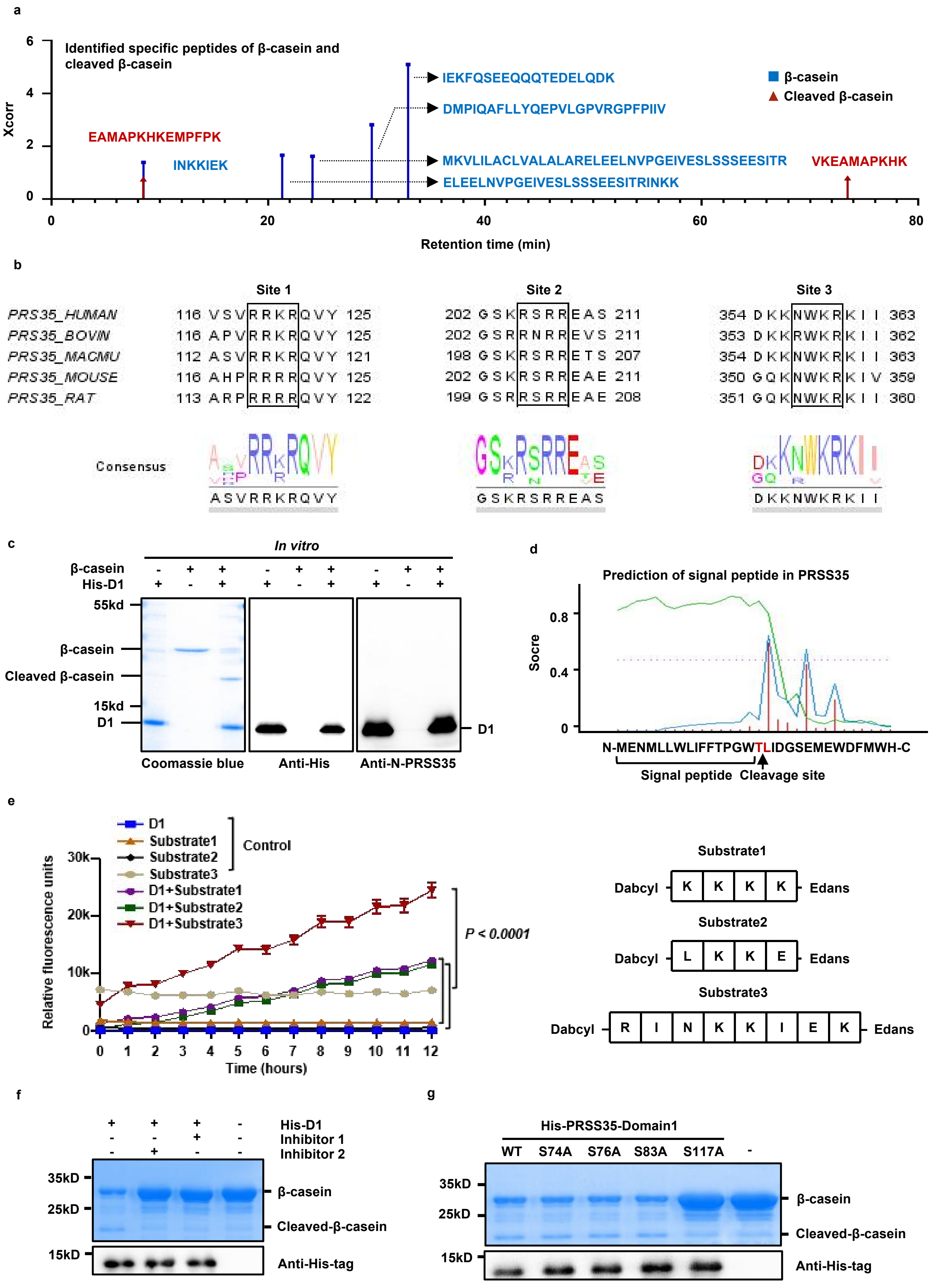

Supplementary Figure 2

Supplementary Fig. 2. PRSS35 activated by FURIN functions as a protease

a, Bands from (2a) were analyzed by mass spectrometry to identify PRSS35 cleavage sites. PRSS35 cleavage of β-casein at K43-K44 was monitored by LC-MS through not identifying peptides containing amino acids in front of K44 in cleaved β-casein band, but identifying full length β-casein band. Identified peptides from full length β-casein and cleaved β-casein were shown.

b, Conserved cleavage motifs by proprotein convertases (PCs) in PRSS35 protein sequence of five species.

c, His-PRSS35-domain1 protein purified from *E. coli* was incubated with β-casein protein at 37°C overnight, followed by SDS-PAGE and coomassie brilliant blue staining (left panel). His-PRSS35-domain1 signal was determined by western blot with anti-His antibody and N-PRSS35 antibody (right panel). D1: PRSS35-domain1.

d, Diagram of prediction of signal peptide in PRSS35.

e, Fluorescent absorbance monitored over time as a measurement of active peptidase activity of PRSS35-domain1 (left panel, n=3 biologically replicates). Sequences of three fluorescent peptides (right panel).

f, *E. coli* purified His-D1 and β-casein protein were incubated with DMSO, serine protease inhibitor 1 (PMSF) or serine protease inhibitor 2 (cocktails) at 37°C overnight, followed by SDS-PAGE and coomassie brilliant blue staining. His-D1 signal was determined by western blot with anti-His antibody. D1: PRSS35 domain1, Serine protease inhibitor 2 (cocktails) included Aprotinin, Bestatin， Leupetin, Pepstatin, PMSF, E-64, and Phosphoramidon.

g, *E. coli* purified wild type His-D1 or mutated His-D1 protein (serine to alanine mutation) as indicated was incubated with β-casein protein at 37°C overnight, followed by SDS-PAGE and coomassie brilliant blue staining. wild type His-D1 and His-D1-mutation signals were determined by western blot with anti-His antibody.

Data are presented as the mean ± s.d. (e). Statistical significance was determined by two-way ANOVA (e). The blotting experiments were repeated at least three times with biological replicates (c, f, g). Source data are provided as a Source Data file.

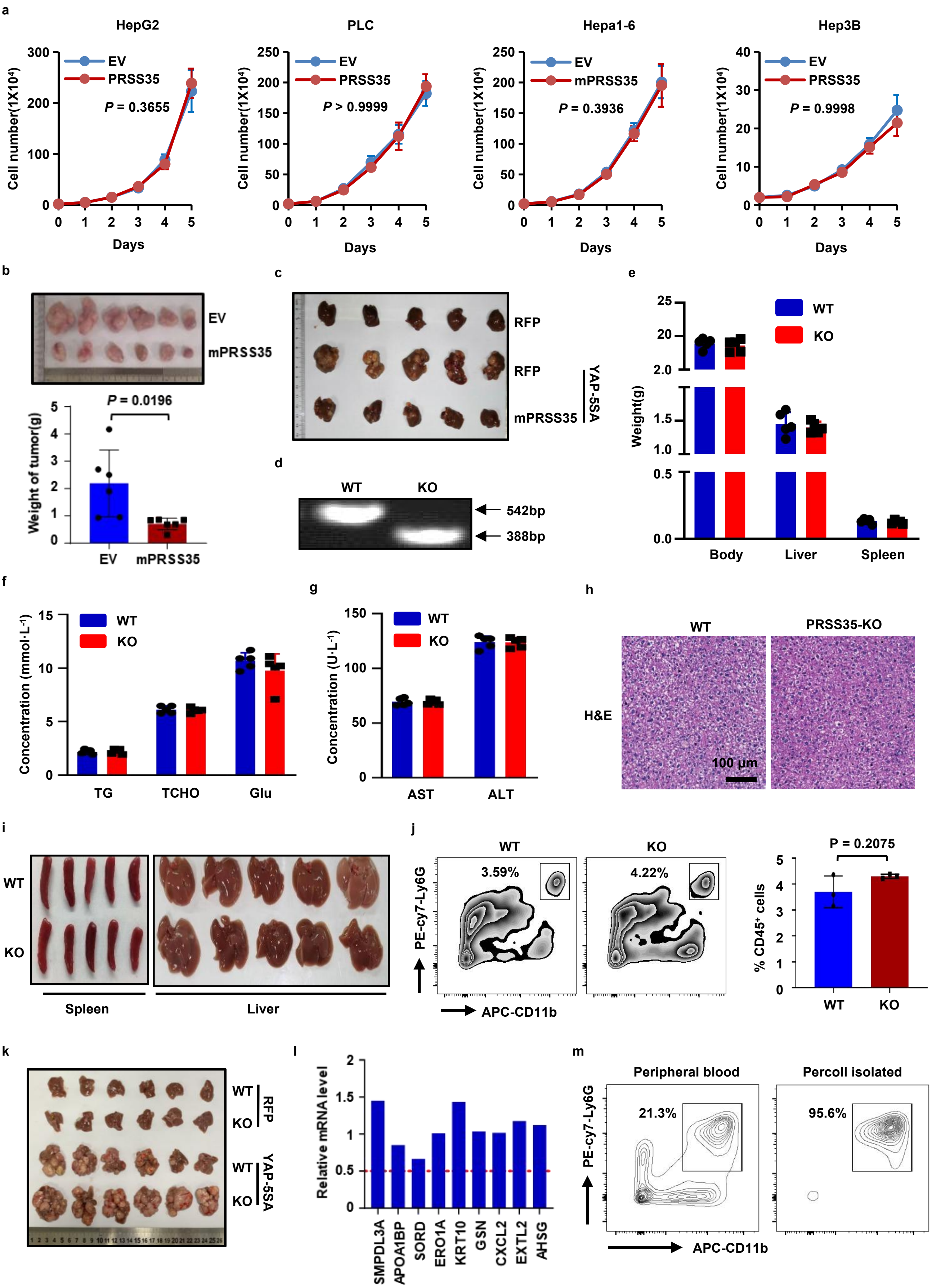

Supplementary Figure 3

Supplementary Fig. 3. PRSS35 suppresses neutrophil migration by degrading CXCL2

a, Growth curves of HepG2, PLC, Hepa1-6, and Hep3B cells stably expressing EV or PRSS35 (mPRSS35). Cell numbers were determined by trypan blue counting (n=5 biologically replicates).

b, Equal numbers of Hepa1-6 cells overexpressing EV or Flag-mPRSS35 were injected subcutaneously into C57BL/6J mice. Tumor weight was measured at 35 days after injection (n=6 biologically replicates).

c, Plasmids expressing YAP-5SA alone or YAP-5SA plus mPRSS35 together with PB transposase plasmids were delivered into ICR mice by hydrodynamic injection. YAP-5SA induced liver tumorigenesis was analyzed approximately 100 days after injection. RFP served as a control. Livers were harvested and imaged at the end of the experiment.

d, Geno-typing result of *mPRSS35* knockout mice. 154bp was deleted in *mPRSS35* knockout mice.

e, Body, liver and spleen weight of WT and *PRSS35*-KO mice (n=5 biologically replicates).

f, Triglyceride (TG), total cholesterol (TCHO) and glucose (Glu) in serum of WT and *PRSS35*-KO mice (n=5 biologically replicates).

g, Aspartic transaminase (AST) and alanine aminotransferase (ALT) concentrations in serum of WT and *PRSS35*-KO mice (n=5 biologically replicates).

h, Hematoxylin staining (H&E) of liver tissues from WT and *PRSS35*-KO mice.

i, Photograph of spleen and liver of WT and *PRSS35*-KO mice.

j, The neutrophil infiltration in the liver tissues of WT and *PRSS35*-KO mice (n=3 biologically replicates).

k, Plasmids expressing YAP-5SA or RFP together with PB transposase plasmids were delivered into *mPRSS35*-KO or WT C57BL/6J mice by hydrodynamic injection (n=6 in each group). YAP-5SA induced liver tumorigenesis was analyzed approximately 100 days after injection. Livers were harvested and imaged at the end of the experiment.

l, mRNA levels of the possible PRSS35-degrading substrates detected by transcriptomics.

m, Validation of the purity of isolated neutrophils from peripheral blood of healthy C57BL/6J mice by flow cytometry.

Data are presented as the mean ± s.d. (a, b, e, f, g, j). Statistical significance was determined by two-way ANOVA (a, b, j). Source data are provided as a Source Data file.

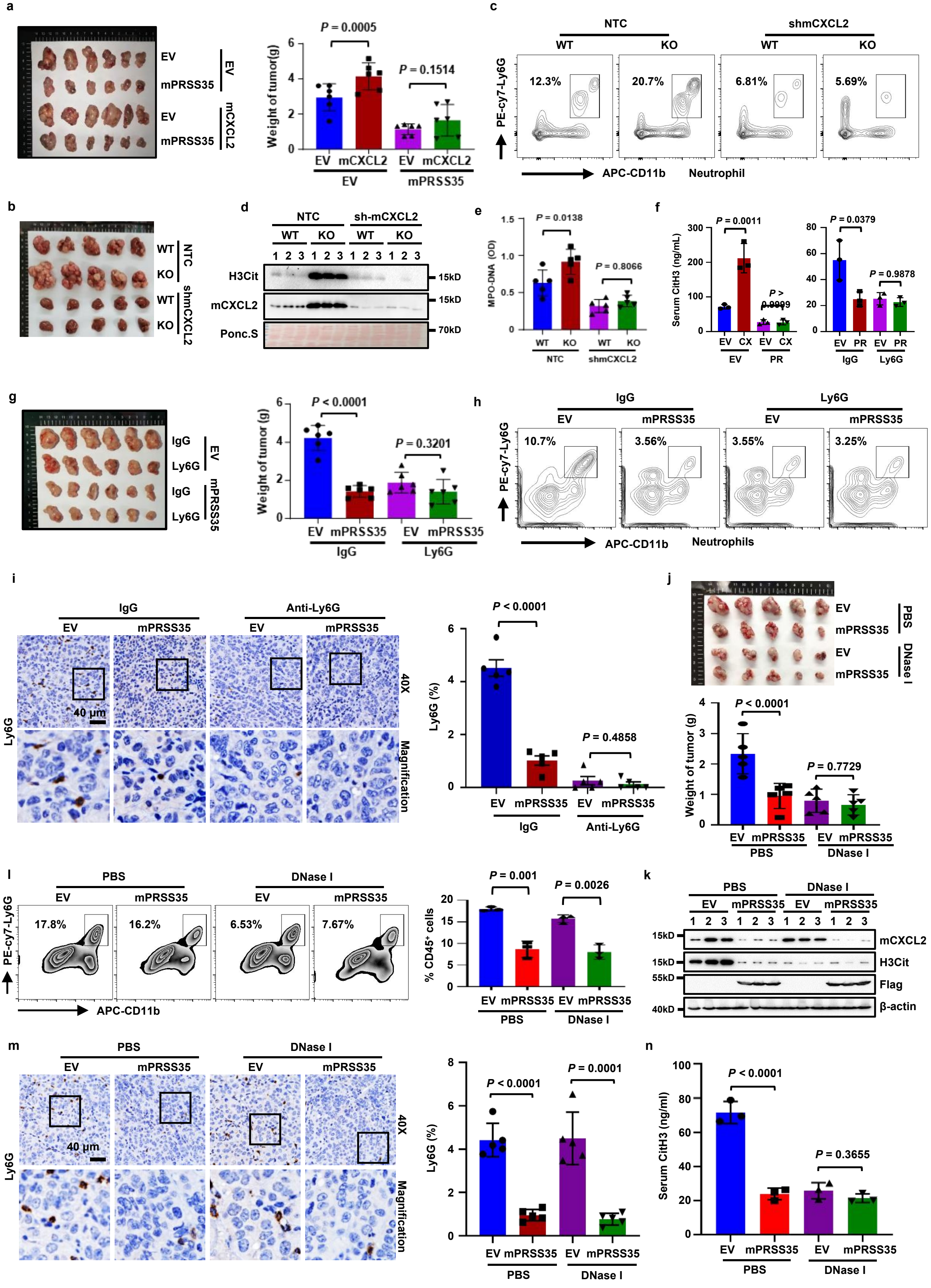

Supplementary Figure 4

o

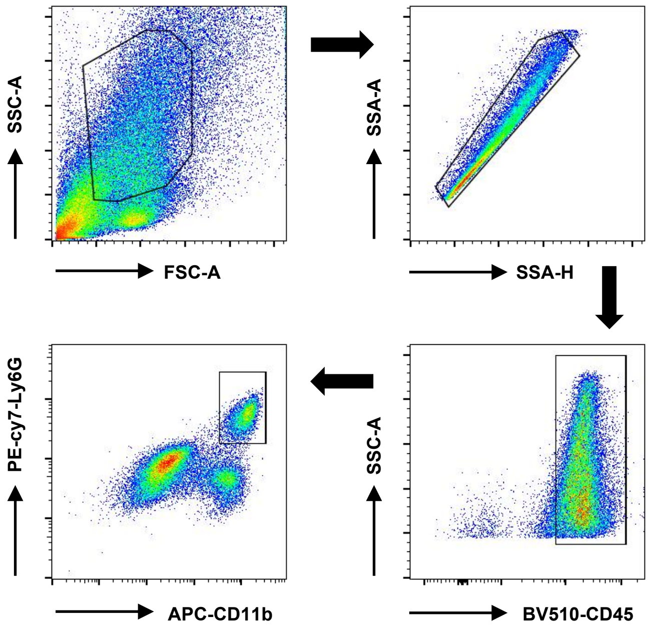

p

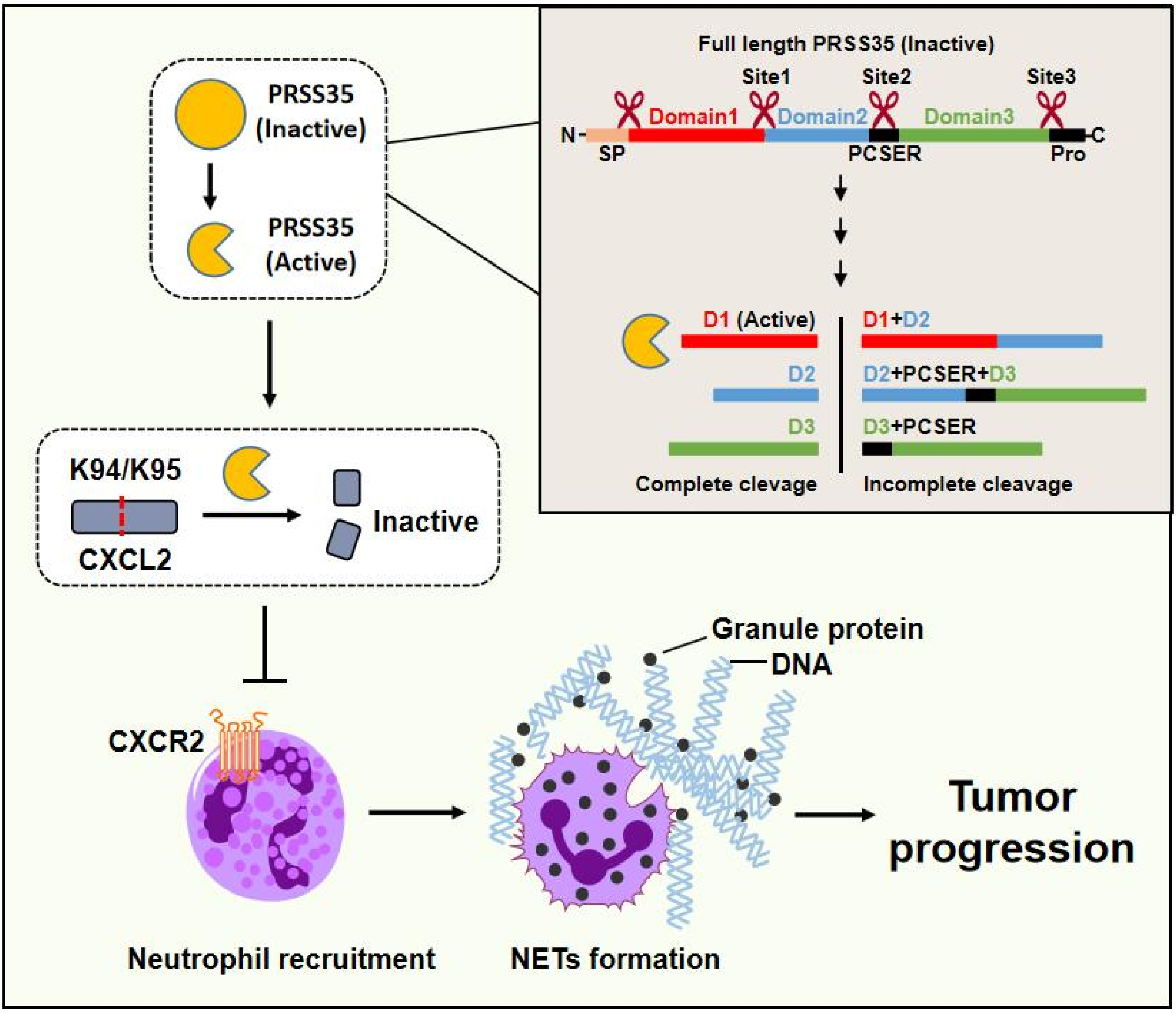

Supplementary Figure 4

Supplementary Fig. 4. PRSS35 inhibits HCC development by suppressing CXCL2-mediated neutrophil NETs formation

a, Hepa1-6 cells stably expressing EV, mCXCL2, Flag-mPRSS35, or mCXCL2 plus Flag-mPRSS35 were injected subcutaneously into C57BL/6J mice. Photograph showed tumors at the end of experiment (left panel). Tumors were extracted and weighted at the end of the experiment (right panel, n=6 biologically replicates).

(b, c, d, e) Plasmids expressing YAP-5SA alone or YAP-5SA plus mCXCL2 shRNAs together with plasmids expressing PB transposase were delivered into *mPRSS35*-KO or WT C57BL/6J mice by hydrodynamic injection. YAP-5SA induced liver tumorigenesis was analyzed approximately 100 days after injection. **b,** Photograph showed tumors at the end of experiment. **c,** Representative FACS analysis of neutrophil in tumors was shown. **d,** Western blot analysis of mCXCL2 and H3Cit protein levels in tumor lysates. Ponceau staining served as a loading control. **e,** Serum NETs levels in mice with HCC *in situ* were measured by MPO-DNA ELISA kit (n=5 biologically replicates).

f, Serum NETs levels were measured by mouse H3Cit ELISA kit using the sera from the mouse experiment in Figure 4d (left panel, n=3 biologically replicates) and Figure 4k (right panel, n=3 biologically replicates).

(g, h, i) Hepa1-6 cells stably expressing EV or Flag-mPRSS35 were injected subcutaneously into C57BL/6J mice, followed by intratumor and peritumoral injection of Ly6G antibody or IgG antibody nine days later. **g,** Photograph showed tumors at the end of experiment (left panel). Tumors were extracted and weighted at the end of the experiment (right panel, n=6 biologically replicates). **h,** Representative FACS analysis of neutrophil in tumors was shown. **i,** Representative IHC analysis of Ly6G expression in tumors (left panel). Statistical quantification of Ly6G-positive area/total area in IHC assay (right panel, n=5 biologically replicates).

j, Hepa1-6 cells stably expressing control empty vector (EV) or Flag-mPRSS35 were injected subcutaneously into C57BL/6J mice, followed by daily intraperitoneal injection of DNase I five days later. Photograph showed tumors at the end of the experiment (upper panel). Tumors were extracted and weighted at the end of the experiment (lower panel, n=5 biologically replicates).

k, Western blot analysis of mCXCL2, Flag-mPRSS5 and H3Cit protein levels in tumors. β-actin served as a loading control.

l, The neutrophils in tumors was detected by Flow cytometry (n=3 biologically replicates).

m, Representative IHC images (left panel) and quantification (right panel, n=5 biologically replicates) of neutrophils in the tumors using anti-Ly6G staining.

n, Serum NETs levels were measured by H3Cit ELISA kit ((n=3 biologically replicates).

o, FACS sequential gating strategies for FACS experiments in Figure 4c, Extended Figure 3j, m, Extended Figure 4c, h.

p, Working model of PRSS35 suppressing HCC progression.

Data are presented as the mean ± s.d. (a, e, f, g, i, j, l, m, n). Statistical significance was determined by two-way ANOVA (a, e, f, g, j, l, n) or two-tailed unpaired Student's t-test (i, m). The blotting experiments were repeated at least three times with biological replicates (d, k). Source data are provided as a Source Data file.

Supplementary Table 1. Clinicopathological characteristics of clinical samples and expression profile of PRSS35 in liver cancer

Characteristics	Number of cases (%)
Age (years)	
≤50	95（60.1）
>50	63（39.9）
AFP	
≤400ug/ul	97（61.4）
>400ug/ul	61（38.6）
Gender	
Female	17（10.8）
Male	141（89.2）
Tumor size	
≤3 cm	21（13.3）
>3 cm	137（86.7）
Tumor node	
≤1	120（75.9）
>1	38（24.1）
HBsAg	
negative	38（24.1）
positive	120（75.9）
Cirrhosis	
Absent	34（21.5）
Present	124（78.5）
Vascular Invasion	
Absent	98（62.0）
Present	60（38.0）
Vital status	
Alive	53（33.5）
Dead	105（66.5）
Clinical stage	
I	12（7.6）
II	92（58.2）
III	38（24.1）
IV	16（10.1）
T classification	
I	12（7.6）
II	92（58.2）
III	38（24.1）
IV	16（10.1）
N classification	
N0	2（1.3）
N1	156（98.7）
M classification	
M0	1（0.6）
M1	157（99.4）
PRSS35	
Low expression	81（51.3）
High expression	77（48.7）

Abbreviationas: HBsAg, hepatitis B surface antigen; AFP, alpha fetoprotein.

Supplementary Table 2. Correlation between PRSS35 expression and clinicopathological Characteristics of liver cancer patients

Variable	Total	PRSS35 expression		Chi-square test
		low	high	p-value
Age (years)				
≤50	63	37	26	
>50	95	44	51	0.126
Gender				
Female	17	12	5	
Male	141	69	72	0.092
Tumor node				
≤1	120	63	57	
>1	38	18	20	0.581
Tumor size（cm）				
≤3	21	8	13	
>3	137	73	64	0.195
Vascular Invasion				
Absent	98	53	45	
Present	60	28	32	0.366
AFP（ug/ul）				
≤400	97	51	46	
>400	61	30	31	0.678
HBsAg				
negative	38	17	21	
positive	120	64	56	0.356
Cirrhosis				
Absent	34	17	17	
Present	124	64	60	0.868
Vital status				
Alive	53	25	28	
Dead	105	56	49	0.464
Clinical stage				
Ⅰ	12	0	12	
Ⅱ-Ⅳ	146	81	65	<0.001

Abbreviationas: HBsAg, hepatitis B surface antigen; AFP, alpha fetoprotein.

Supplementary Table 3. Spearman analysis of the correlation between PRSS35 and clinicopathological characteristics

Variables	PRSS35 expression level	
	Spearman Correlation	p-Value
Survival time	0.312	<0.001
Vital status	-0.023	0.773
Clinical stage	-0.15	0.06
Tumor size	-0.106	0.184

Supplementary Table 4. Oligonucleotide sequences of shRNA

Oligonucleotide sequence of shRNAs	
**Genes**	**Sequence**
**shFURIN-1**	**CCGGCCTGTCCCTCTAAAGCAATAACTCGAGTTATTGCTTTAGAGGGACAGGTTTTTG**
**shFURIN-2**	**CCGGCCACATGACTACTCCGCAGATCTCGAGATCTGCGGAGTAGTCATGTGGTTTTTG**
**shCXCL2-1（Mouse）**	**CCGGGCCAAGGGTTGACTTCAAGAACTCGAGTTCTTGAAGTCAACCCTTGGCTTTTTG**
**shCXCL2-2（Mouse）**	**CCGGCCACTCTCAAGGGCGGTCAAACTCGAGTTTGACCGCCCTTGAGAGTGGTTTTTG**
**shCXCL2-3（Mouse）**	**CCGGACTGAACAAAGGCAAGGCTAACTCGAGTTAGCCTTGCCTTTGTTCAGTTTTTG**

Supplementary Table 5. The information of serum samples from 149 liver cancer patients and 73 healthy donors

Patient	Gender	Age (years)	Tumor	Patient	Gender	Age (years)	Tumor	Patient	Gender	Age (years)	Tumor
1	female	67	HCC	51	male	63	HCC	101	male	56	HCC
2	female	82	HCC	52	male	26	HCC	102	male	75	HCC
3	male	38	HCC	53	male	41	HCC	103	female	44	HCC
4	male	45	HCC	54	male	63	HCC	104	female	45	HCC
5	male	48	HCC	55	male	56	HCC	105	female	65	HCC
6	female	52	HCC	56	male	54	HCC	106	female	52	HCC
7	male	62	HCC	57	male	66	HCC	107	male	54	HCC
8	male	71	HCC	58	male	74	HCC	108	male	78	HCC
9	male	40	HCC	59	male	58	HCC	109	male	55	HCC
10	male	74	HCC	60	male	64	HCC	110	female	41	HCC
11	male	48	HCC	61	male	47	HCC	111	female	35	HCC
12	female	53	HCC	62	male	51	HCC	112	female	71	HCC
13	male	55	HCC	63	male	43	HCC	113	female	63	HCC
14	male	71	HCC	64	female	63	HCC	114	male	62	HCC
15	male	56	HCC	65	male	76	HCC	115	male	59	HCC
16	male	45	HCC	66	male	41	HCC	116	female	57	HCC
17	male	67	HCC	67	male	58	HCC	117	male	23	HCC
18	female	69	HCC	68	female	50	HCC	118	male	49	HCC
19	female	70	HCC	69	male	56	HCC	119	male	46	HCC
20	male	55	HCC	70	male	67	HCC	120	male	54	HCC
21	female	46	HCC	71	male	70	HCC	121	male	46	HCC
22	male	68	HCC	72	male	64	HCC	122	male	56	HCC
23	male	70	HCC	73	male	49	HCC	123	female	54	HCC
24	male	54	HCC	74	male	85	HCC	124	male	46	HCC
25	male	73	HCC	75	male	67	HCC	125	female	50	HCC
26	male	70	HCC	76	male	63	HCC	126	female	57	HCC
27	female	61	HCC	77	male	71	HCC	127	female	46	HCC
28	female	58	HCC	78	male	76	HCC	128	female	70	HCC
29	female	56	HCC	79	male	64	HCC	129	male	48	HCC
30	male	59	HCC	80	male	61	HCC	130	female	66	HCC
31	male	53	HCC	81	male	47	HCC	131	male	66	HCC
32	male	73	HCC	82	female	72	HCC	132	male	48	HCC
33	female	41	HCC	83	female	74	HCC	133	male	55	HCC
34	female	61	HCC	84	male	66	HCC	134	female	72	HCC
35	male	51	HCC	85	female	54	HCC	135	male	54	HCC
36	male	55	HCC	86	male	64	HCC	136	male	68	HCC
37	female	47	HCC	87	male	70	HCC	137	female	62	HCC
38	male	86	HCC	88	male	82	HCC	138	male	62	HCC
39	male	48	HCC	89	male	61	HCC	139	female	73	HCC
40	female	54	HCC	90	female	65	HCC	140	male	62	HCC
41	male	76	HCC	91	male	62	HCC	141	male	54	HCC
42	female	66	HCC	92	female	68	HCC	142	male	50	HCC
43	male	55	HCC	93	male	45	HCC	143	male	49	HCC
44	male	54	HCC	94	female	59	HCC	144	male	65	HCC
45	male	60	HCC	95	male	82	HCC	145	male	56	HCC
46	male	59	HCC	96	male	45	HCC	146	female	56	HCC
47	male	42	HCC	97	male	56	HCC	147	male	63	HCC

48	male	66	HCC	98	male	61	HCC	148	female	35	HCC
49	male	47	HCC	99	male	66	HCC	149	female	66	HCC
50	male	62	HCC	100	female	78	HCC				

Supplementary Table 5 continued

Healthy donor	Gender	Age(years)	Healthy donor	Gender	Age(years)
1	male	47	38	male	35
2	female	31	39	male	42
3	female	31	40	male	37
4	male	41	41	male	36
5	female	27	42	male	38
6	female	29	43	female	34
7	male	28	44	female	48
8	female	29	45	male	31
9	female	49	46	male	43
10	female	45	47	female	54
11	male	32	48	male	45
12	female	47	49	female	50
13	male	47	50	male	58
14	male	29	51	female	30
15	female	19	52	female	50
16	female	32	53	male	51
17	female	56	54	female	51
18	female	52	55	female	43
19	male	27	56	male	53
20	male	28	57	female	38
21	female	42	58	female	24
22	female	38	59	male	61
23	male	36	60	male	66
24	female	35	61	female	34
25	female	38	62	male	71
26	female	47	63	male	66
27	male	26	64	male	35
28	male	86	65	male	84
29	male	35	66	male	48
30	male	39	67	female	37
31	female	42	68	male	53
32	female	47	69	male	24
33	male	36	70	male	33
34	male	64	71	male	32
35	male	44	72	female	31
36	male	37	73	female	37
37	female	55			

Source data-Supplementary Figure 1

b

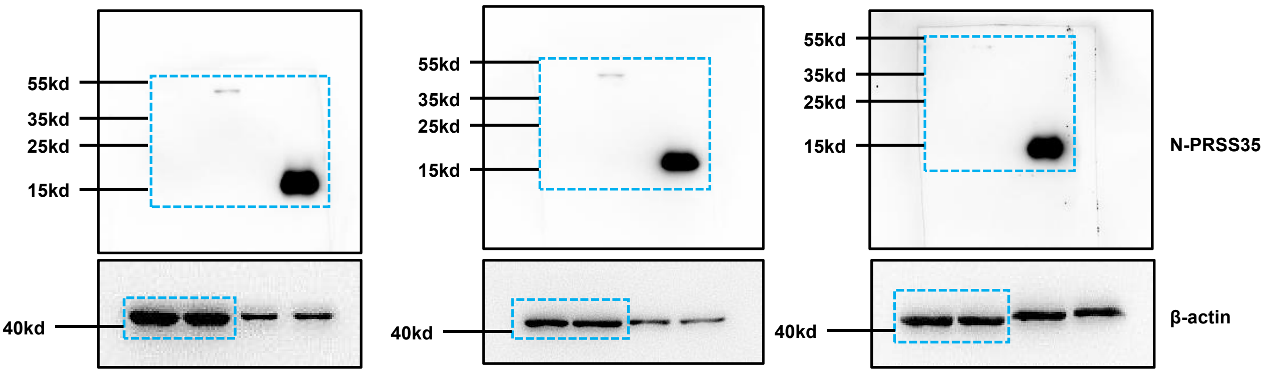

d

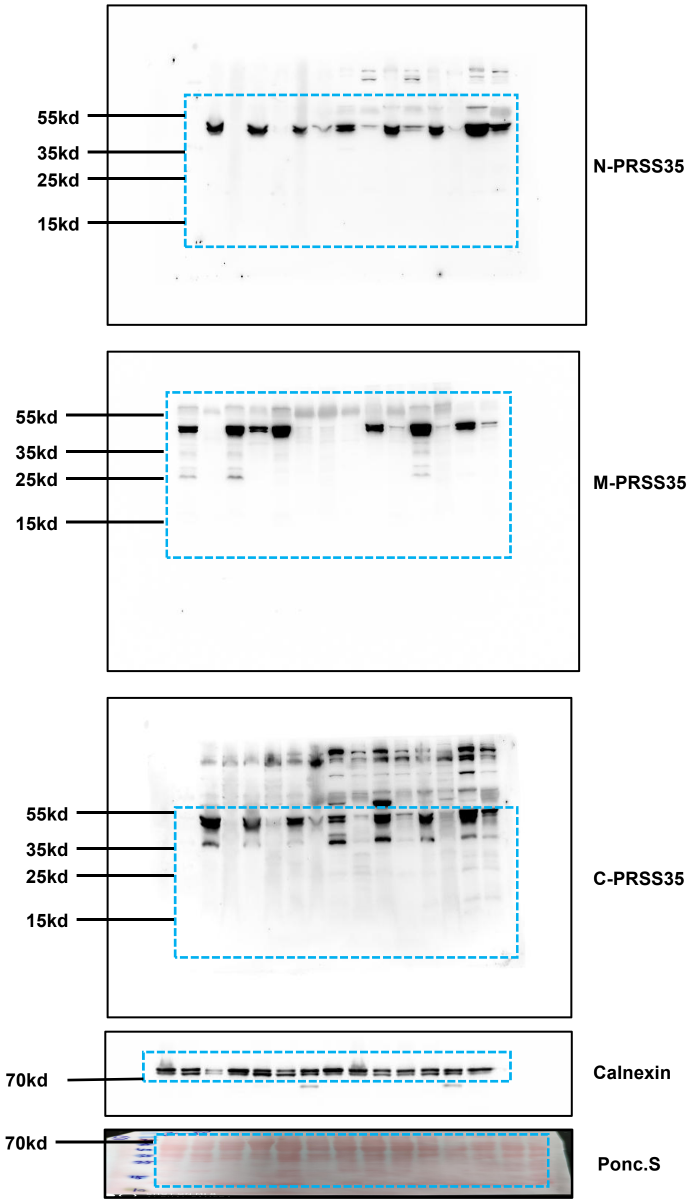

i

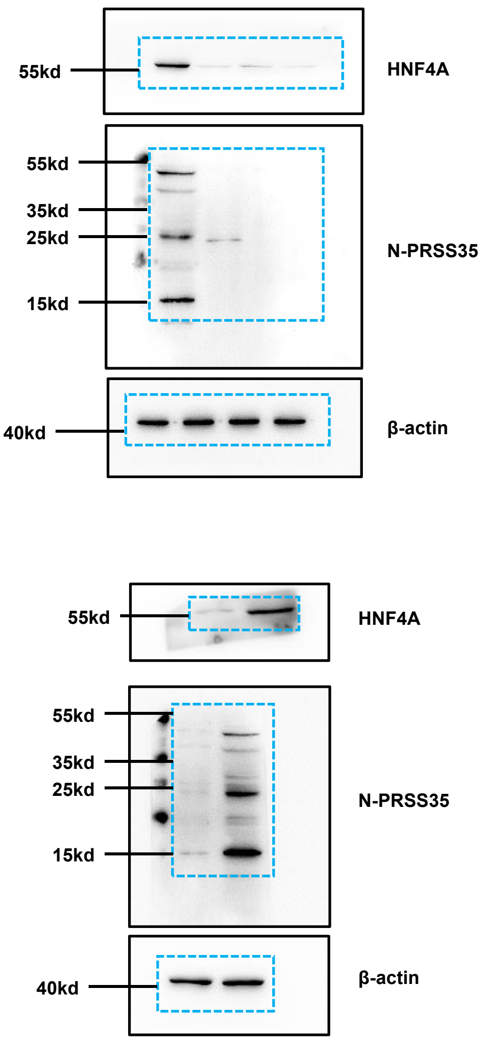

Source data-Supplementary Figure 2

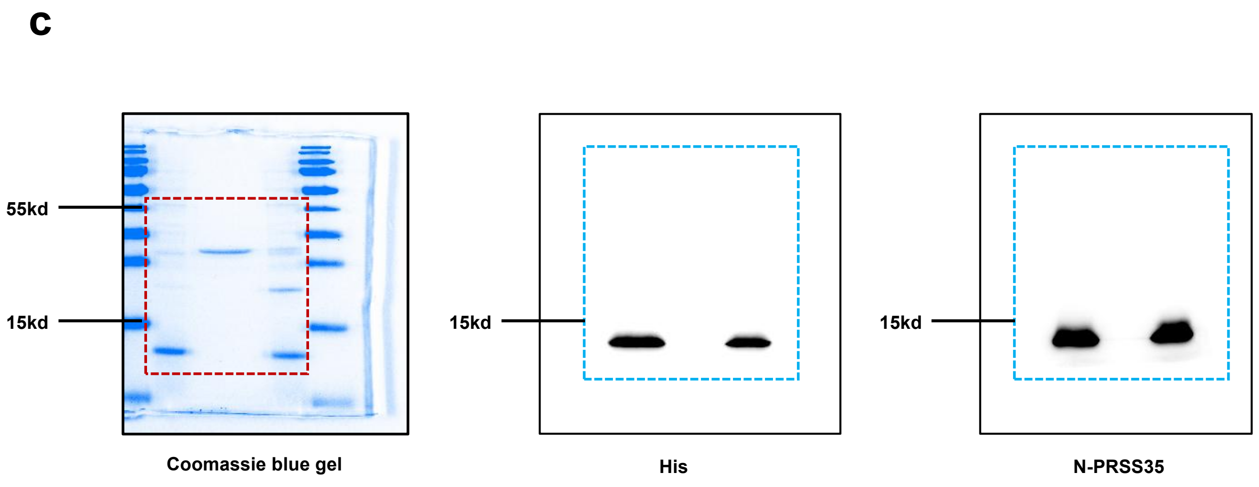

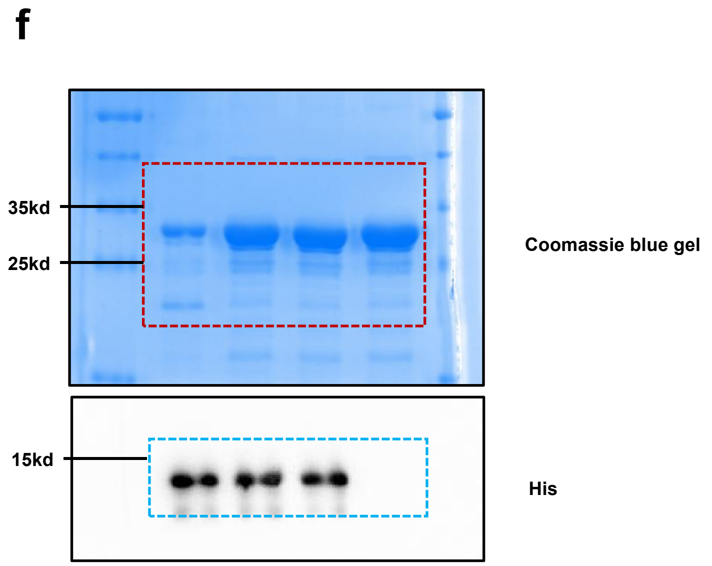

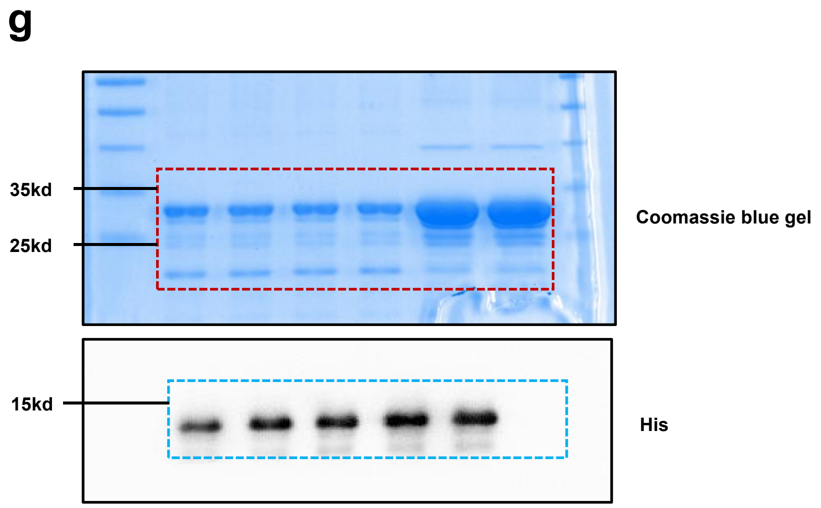

Source data-Supplementary Figure 3

d

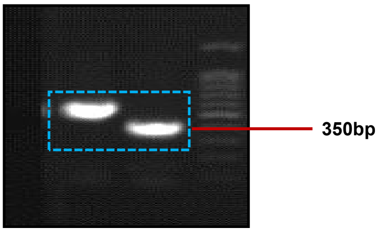

Source data-Supplementary Figure 4

d

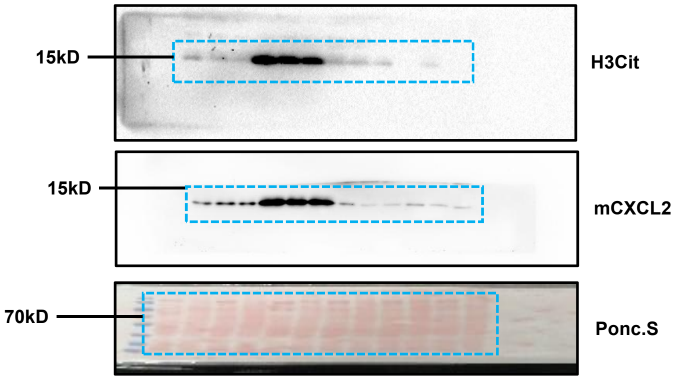

k

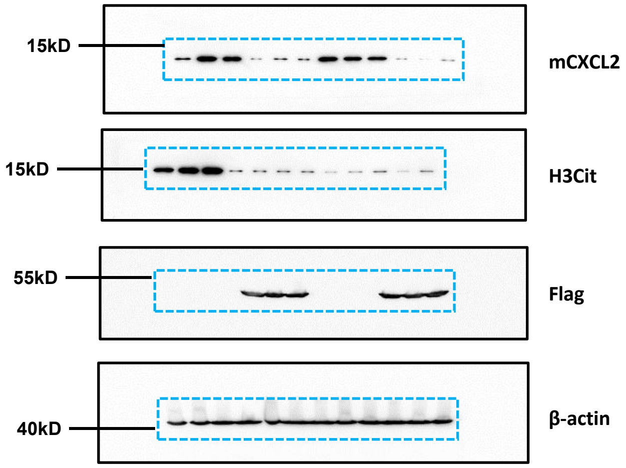

Supplement: Supplementary file 1 — Supplementary Information [file 41467_2023_37227_MOESM1_ESM.pdf]
