# Supplementary material for: Secreted protease PRSS35 suppresses hepatocellular carcinoma by disabling CXCL2-mediated neutrophil extracellular traps
Source: Nat Commun. 2023 Mar 18;14:1513. doi: 10.1038/s41467-023-37227-z (PMC10024721; doi:10.1038/s41467-023-37227-z)
Supplement: Supplementary file 3 — Description of Additional Supplementary Files [file 41467_2023_37227_MOESM3_ESM.pdf]

Description of Additional Supplementary Files

File Name: Supplementary Data 1
Description: Cleavage windows (New-born peptide terminals) producedbyPRSS35 in 22 potential substrates.

File Name: Supplementary Data 2
Description: The detailed mass spectrum (MS) information of label-free proteomic analysis of the secreted protein profiles of human PLC liver cancer cells and human THLE3 hepatocytes.

File Name: Supplementary Data 3
Description: Mass spectrometry (MS) information of the small molecular bands detected by SDS-PAGE in Fig. 1d.
