# Supplementary material for: Secreted protease PRSS35 suppresses hepatocellular carcinoma by disabling CXCL2-mediated neutrophil extracellular traps
Source: Nat Commun. 2023 Mar 18;14:1513. doi: 10.1038/s41467-023-37227-z (PMC10024721; doi:10.1038/s41467-023-37227-z)
Supplement: Supplementary file 7 — Reporting Summary [file 41467_2023_37227_MOESM7_ESM.pdf]

nature portfolio

Corresponding author(s): Huafeng Zhang

Last updated by author(s): Feb 21, 2023

Reporting Summary

Nature Portfolio wishes to improve the reproducibility of the work that we publish. This form provides structure for consistency and transparency in reporting. For further information on Nature Portfolio policies, see our Editorial Policies and the Editorial Policy Checklist.

Statistics

For all statistical analyses, confirm that the following items are present in the figure legend, table legend, main text, or Methods section.

n/a	Confirmed	
☐	☒	The exact sample size (*n*) for each experimental group/condition, given as a discrete number and unit of measurement
☐	☒	A statement on whether measurements were taken from distinct samples or whether the same sample was measured repeatedly
☐	☒	The statistical test(s) used AND whether they are one- or two-sided<br>*Only common tests should be described solely by name; describe more complex techniques in the Methods section.*
☒	☐	A description of all covariates tested
☐	☒	A description of any assumptions or corrections, such as tests of normality and adjustment for multiple comparisons
☐	☒	A full description of the statistical parameters including central tendency (e.g. means) or other basic estimates (e.g. regression coefficient) AND variation (e.g. standard deviation) or associated estimates of uncertainty (e.g. confidence intervals)
☐	☒	For null hypothesis testing, the test statistic (e.g. *F*, *t*, *r*) with confidence intervals, effect sizes, degrees of freedom and *P* value noted<br>*Give P values as exact values whenever suitable.*
☒	☐	For Bayesian analysis, information on the choice of priors and Markov chain Monte Carlo settings
☒	☐	For hierarchical and complex designs, identification of the appropriate level for tests and full reporting of outcomes
☒	☐	Estimates of effect sizes (e.g. Cohen's *d*, Pearson's *r*), indicating how they were calculated

Our web collection on statistics for biologists contains articles on many of the points above.

Software and code

Policy information about availability of computer code

Data collection	Q-Exactive-plus mass spectrometer (Thermo Fisher Scientific) was used to collect all proteomics data.<br>Illumina Hiseq6000 platform (Novogene, Tianjing, China) was used to collect RNA-seq data; Reads were aligned to the human genome hg19 (https://www.ncbi.nlm.nih.gov/assembly/GCF_000001405.13/).<br>Images of IHC was acquired with a Zeiss AxioImager Z1(Carl Zeiss AG).
Data analysis	Proteome Discovery version 2.2 with Seaquest HT (v1.17) search engine and Maxquant version 1.5.8.5 with Andromeda search engine (without version number) were used to analyze proteomics data.<br>TopHat2 v2.1.0, Cuffdiff v2.2.1 and Integrative Genomics Viewer (IGV 2.10.3) were used to analyze RNA-seq data; Gene expression analysis was performed using the DEGSeq R package (1.26.0).<br>HistoQuest analysis software (Version 6.0) was used to analyze Images quantification of IHC.<br>Flowjo v10 and BD FACSDiva Software (V8.03) were used to analyze FACS data.

For manuscripts utilizing custom algorithms or software that are central to the research but not yet described in published literature, software must be made available to editors and reviewers. We strongly encourage code deposition in a community repository (e.g. GitHub). See the Nature Portfolio guidelines for submitting code & software for further information.

Data

Policy information about availability of data

All manuscripts must include a data availability statement. This statement should provide the following information, where applicable:

- Accession codes, unique identifiers, or web links for publicly available datasets
- A description of any restrictions on data availability
- For clinical datasets or third party data, please ensure that the statement adheres to our policy

RNA-seq data generated in this study have been deposited in the Gene Expression Omnibus under accession code GSE201311 (https://www.ncbi.nlm.nih.gov/geo/query/acc.cgi?acc=GSE201311). The mass spectrometry proteomics data generated in this study have been deposited to the ProteomeXchange Consortium via the PRIDE83 partner repository with the dataset identifier PXD033437 (https://www.ebi.ac.uk/pride/archive/projects/PXD033437) and PXD033579 (https://www.ebi.ac.uk/pride/archive/projects/PXD033579). All other data supporting the results of this study can be obtained from the corresponding author upon reasonable request.

Human research participants

Policy information about studies involving human research participants and Sex and Gender in Research.

Reporting on sex and gender	Our findings apply to both genders. Gender was considered in our study design. We have collected the gender informaiton of participants.
Population characteristics	The serum samples from 149 liver cancer patients and 73 healthy individuals were obtained from the First Affiliated Hospital of University of Science and Technology of China. Information of all 149 patients (No.1-149) and 73 donors (No.1-73) are provided in Methods.<br>Of the 149 liver cancer patients, 45 were women and 104 were men, with 114 patients over the age of 50. Of the 73 healthy individuals,34 were women and 39 were men,with 18 patients over the age of 50.<br>Formalin-fixed,paraffin-embedded primary HCC specimens obtained from 158 patients were randomly selected from the archives of the First Affiliated Hospital of University of Science and Technology of China. Of the 158 HCC patients, 17 were women and 141 were men,with 63 patients over the age of 50.
Recruitment	We used the serum samples of patients diagnosed with HCC for the serum PRSS35 measurement, which were mainly from 2018 to 2019. At the same time, normal tissue samples were obtained from patients who were not diagnosed as HCC from 2018 to 2019.<br>We used the samples of patients diagnosed with HCC for immunohistochemical experiments, which were mainly from 2005 to 2011. At the same time, normal tissue samples were obtained from patients who were not diagnosed as HCC from 2005 to 2011.<br>We used the samples of patients diagnosed with HCC for WB experiments, which were mainly from 2018 to 2019.<br>For use of these clinical materials for research purpose, prior patients' written informed consents and approval from the Institutional Research Ethics Committee of the First Affiliated Hospital of University of Science and Technology of China were obtained. No other biases existed in this study.
Ethics oversight	Ethical approval for the studies was obtained from the Institutional Research Ethics Committee of the First Affiliated Hospital of University of Science and Technology of China.

Note that full information on the approval of the study protocol must also be provided in the manuscript.

Field-specific reporting

Please select the one below that is the best fit for your research. If you are not sure, read the appropriate sections before making your selection.

☒ Life sciences ☐ Behavioural & social sciences ☐ Ecological, evolutionary & environmental sciences

For a reference copy of the document with all sections, see nature.com/documents/nr-reporting-summary-flat.pdf

Life sciences study design

All studies must disclose on these points even when the disclosure is negative.

Sample size	We determined the sample sizes based on preliminary studies in our laboratories or in similarly published research. The samples were enough to be detected and we observed statistical significant difference in replicated independent experiments. For animal experiments, we used 5 or 6 male mice for each group and followed the 3 R's of animal research.
Data exclusions	No data were excluded from the data set.

Replication	Each experiment was repeated at least three times independently with similar results. For animal studies, we used 5 or 6 male mice for each different group, and the statistical significance was shown in figures. We confirmed successful replication for our reported data.
Randomization	Mice were randomly allocated to control group or treatment groups. For in vitro experiments, all samples were analyzed equally with no sub-sampling, and therefore was no requirement for randomization.
Blinding	Data collection and analysis were not performed blind to the conditions of the experiments. In the survival analysis of HCC patients, the investigators were blinded for the clinical information of each sample prior to immunostaining analysis.

Reporting for specific materials, systems and methods

We require information from authors about some types of materials, experimental systems and methods used in many studies. Here, indicate whether each material, system or method listed is relevant to your study. If you are not sure if a list item applies to your research, read the appropriate section before selecting a response.

Materials & experimental systems

n/a	Involved in the study
☐	☒ Antibodies
☐	☒ Eukaryotic cell lines
☒	☐ Palaeontology and archaeology
☐	☒ Animals and other organisms
☒	☐ Clinical data
☒	☐ Dual use research of concern

Methods

n/a	Involved in the study
☒	☐ ChIP-seq
☐	☒ Flow cytometry
☒	☐ MRI-based neuroimaging

Antibodies

Antibodies used

Antibodies for western blot:
N-PRSS35 Polyclonal; Therofisher; cat: PA5-58542; lot: UG302804313; 1:500
M-PRSS35 Polyclonal; Custom in Abclonal; 1:500
C-PRSS35 Polyclonal; Custom in Abclonal; 1:500
Furin Polyclonal; Proteintech; cat: 18413-1-AP; lot: 00025330; 1:1000
Mouse CXCL2; R&D; cat: AF-452; lot: UR0520021
CXCL2 Polyclonal; Abclonal; cat: A12639; lot: 0054770201; 1:500
Histone H3 (citrulline R2); Abcam; cat: ab176843; clone name: EPR17703; lot: GR3250183-7; 1:500
Calnexin Polyclonal; Proteintech; cat: 10427-2-AP; lot: 00094165; 1:5000
β-Actin Monoclonal; Proteintech; cat: 66009-1-lg; clone name: 2D4H5; lot: 10004156; 1:5000
Flag-M2 Monoclonal; Sigma-Aldrich; cat: F1804; clone name: M2; lot: SLBK1346V; 1:3000
Myc-tag; Proteintech;cat: 60003-2-Ig; lot: 10003656
His-tag Monoclonal; Proteintech; cat: 66005-1-Ig; clone name: 1B7G5; lot: 00104948; 1:5000
HRP-conjugated anti-rabbit secondary antibody; BioRad; cat: 1706515; lot: L005679; 1:10000
HRP-conjugated anti-mouse secondary antibody; BioRad; cat: 1706516; lot: L005680; 1:10000
Antibodies for immunohystochemistry:
C-PRSS35 Polyclonal; Custom in Abclonal; 1:200
Ly6G(mGr1); R&D; cat: MAB1037; clone name: RB6-8C5; lot: FVF0414081; 1:200
Antibodies for flow cytometry:
CD16/32; Biolegend; cat: 101302; clone name: 93; lot: B298973; 1:100
BV510-CD45; Biolegend; cat: 109837; clone name:104; lot: B292995; 1:100
APC-CD11b; Biolegend; cat: 101211; clone name: M1/70; lot: B261577; 1:100
APC-cy7-Gr-1 (Ly6G); Biolegend; cat: 108424; clone name: RB6-8C5; lot: B313782; 1:100
PE-cy7-Gr-1 (Ly6G); Biolegend; cat: 108416; clone name: RB6-8C5; lot: B248638; 1:100
Antibodies for ELISA:
PRSS35 antibodies pair; Custom in Raybiotech
MPO Polyclonal; Proteintech; cat: 22225-1-AP; lot: 00067718
Peroxidase-labelled anti-DNA monoclonal antibody; Roche; cat: 11774425001; clone name: clone MCA-33; lot: 52792300; 1:100;
Antibodies for injection of mice:
Normal Rat IgG; R&D; cat: 6-001-A; lot: WAY1319101
Ly6G(mGr1); Proteintech; cat: 65140-1-Ig; clone name: RB6-8C5; lot: 51000662

Validation

These antibodies have either been validated in published literatures or validated in our lab by Western blot using specific targeting shRNAs or over-expressing vectors. Validation statements were shown on the manufacturer's website, including Proteintech, Abcam, Therofisher, Abclonal, R&D, Biolegend.

Eukaryotic cell lines

Policy information about cell lines and Sex and Gender in Research

Cell line source(s)	human HEK293, HEK293T, THLE3, PLC, HepG2, Hep3B and mouse Hepa 1-6 cells were purchased from ATCC.
Authentication	Cell line identities were confirmed by STR profiling.
Mycoplasma contamination	All cell lines were tested routinely to make sure they are negative for mycoplasma contamination by Mycoplasma PCR detecting method.
Commonly misidentified lines (See ICLAC register)	No commonly misidentified cell lines were used.

Animals and other research organisms

Policy information about studies involving animals; ARRIVE guidelines recommended for reporting animal research, and Sex and Gender in Research

Laboratory animals	4-week-old male mice (C57BL/6J; SLAC ANIMAL COMPANY);<br>4-week-old male nude mice (BALB/c nude mice; Beijing Vital River Laboratory Animal Technology Co., Ltd);<br>4-week-old male mice (ICR mice, SLAC ANIMAL COMPANY)<br>PRSS35−/− mice (C57BL/6J) were generated using CRISPR genome editing (target sequence: 5’-AACGAGGGTACCGCTGCAGC-3’ and 5’-ACTCGGAACAGCAGCGTAAA-3’) and were obtained from the animal facility of the University of Science and Technology of China.<br>All animals were housed at a suitable temperature (22–24 °C) and humidity (40–70%) under a 12/12-h light/dark cycle with unrestricted access to food and water for the duration of the experiment.
Wild animals	No wild animals were used in this study.
Reporting on sex	Our findings apply to both genders. Gender was considered in study design. In this study, we used only male mice.
Field-collected samples	No field-collected samples were used in this study.
Ethics oversight	All animal studies were conducted with approval from the Animal Research Ethics Committee of University of Science and Technology of China.

Note that full information on the approval of the study protocol must also be provided in the manuscript.

Flow Cytometry

Plots

Confirm that:

☒ The axis labels state the marker and fluorochrome used (e.g. CD4-FITC).

☒ The axis scales are clearly visible. Include numbers along axes only for bottom left plot of group (a 'group' is an analysis of identical markers).

☒ All plots are contour plots with outliers or pseudocolor plots.

☒ A numerical value for number of cells or percentage (with statistics) is provided.

Methodology

Sample preparation	For tumor-infiltrating immune cell isolation, Hepa 1-6 tumors were mechanically minced and digested by collagenase D (1mg/ml, sigma) at 37 °C for 30 min. For spontanious liver cancer, minced liver tumors were digested in RPMI-1640 medium supplemented with 0.5 mg/mL Collagenase Type IV (GIBCO), and then dissociated cells were passed through a 70-µm cell strainer (BD) and isolated by 40% Percoll (GE Healthcare) gradient centrifugation at 800 g for 20 min. The isolated TILs were then stained with fluorochrome conjugated antibodies. After a washing step, cells were analyzed on an LSRFortessa flow cytometer (BD Biosciences).<br>To isolate neutrophils from peripheral blood of mice, whole blood was collected via cardiac puncture (1ml per animal) and suspended in HBSS (2ml per animal) with 15mM EDTA. After centrifugation (400g, 10min, 4℃), white cells were resuspended in 2ml HBSS with 2mM EDTA. Then, the cells were centrifuged (1500g, 30min, room temperature) in a three-layer Percoll gradient (78%, 69%, and 52%) without braking. Neutrophils enriched in the interface of 69% and 78% layers were confirmed to be of > 95% purity by SRFortessa flow cytometer (BD Biosciences).
Instrument	LSRFortessa flow cytometer (BD Biosciences).
Software	BD FACSDiva Software (v8.03) was used to collect data, while FlowJo v10 was used to analyze flow cytometry data.

Cell population abundance	10,000 cells were analyzed for each sample. For sorting cell, the purity of cells was confirmed within post-sort fractions and regularly maintained > 95%.
Gating strategy	For all experiments, cells were first gated by FSC/SSC to exclude debris, followed by gating SSC-A and SSC-H to eliminate non-singlets. Then, target cell population for further analysis were gated by cell surface marker (e.g. CD45, CD11b, Ly6G). Isotype control antibodies were used as negative controls.

☒ Tick this box to confirm that a figure exemplifying the gating strategy is provided in the Supplementary Information.
